# 5 Admission Frailty Is Associated with Acute Respiratory Failure and Mortality in Burn Patients > 50

**DOI:** 10.1093/jbcr/irac012.009

**Published:** 2022-03-23

**Authors:** Colette Galet, Kevin Lawrence, Kathleen S Skipton Romanowski, Dionne Skeete, Neil Mashruwala

**Affiliations:** University of Iowa, Iowa City, Iowa; Naval Medical Center San Diego, Iowa City, Iowa; UC Davis, Sacramento, California; University of Iowa, Iowa City, Iowa; Carle Foundation Hospital, Urbana, Illinois

## Abstract

**Introduction:**

Pre-injury frailty has been shown to predict mortality of older burn patients. Herein, we assessed the utility of the Canadian Study of Health and Aging Clinical Frailty Scale (CSHA-CFS) to predict burn-specific outcomes. We hypothesize that frail patients are at greater risk for complications such as graft loss, acute respiratory failure, and acute kidney injury and will require increased healthcare support at discharge.

**Methods:**

This is a retrospective cohort study. Patients 50 years and older admitted to our Institution for burn injuries between July 2009 and June 2019 were included. Patients with inhalation injury only, no data on total burn surface area, or for whom medical history was incomplete were excluded. Demographics; comorbidities; pre-injury functional status; admission, injury, and hospitalization information; complications (graft loss, acute respiratory failure, and acute kidney disease (AKI)); mortality, and discharge disposition were collected. Patients were scored on the CSHA-CFS based on pre-admission health and functional status. The frail and non-frail groups were compared. Multivariate analyses were performed to assess the association between admission frailty and outcomes. P < 0.05 was considered significant.

**Results:**

We included 851 patients, 697 were not frail and 154 were frail. Frail patients were significantly older (66.1 ± 10.8 vs. 63.5 ± 10.9, p = 0.002), more likely Caucasian (98.1% vs. 91%, p = 0.027) and to have suffered flame burn injuries (68.8% vs. 59.8%, p < 0.001). Frail patients had a lower %TBSA (4.4 ± 8.1% vs. 10.1 ± 13.1, p < 0.001) but were more likely to stay longer in hospital relative to %TBSA (3.6 ± 6.7 vs. 1.9 ± 3.1, p < 0.001). Frail patients were less likely to have had skin graft procedures (27.3% vs. 57.4, p < 0.001). On multivariate analysis, controlling for age, sex, race, mechanism of injury, %TBSA, 2^nd^ degree and 3^rd^ degree burn surface, inhalation injury, frailty was associated with acute respiratory failure (OR = 2.599 [1.460-4.628], p = 0.001). Frailty was also associated with mortality (OR = 6.915 [2.455-19.980]; p < 0.001) when controlling for the same variables as well as acute respiratory failure and AKI. Frailty was also associated with discharge to home with healthcare services (OR = 2.678 [1.491-4.809], p = 0.001), to SNF, rehabilitation, or long-term acute care facilities (OR = 3.572 [1.933-6.602], p < 0.001), and to hospice (OR = 5.759 [1.519-21.827], p = 0.010) when compared to home without healthcare services.

**Conclusions:**

Frailty is associated with increased risk of acute respiratory failure, mortality, and requiring increased healthcare support post-discharge. Our data suggest frailty as a tool to predict morbidity and mortality as well as for goals of care discussions for the burn patient.

